# Creatine and low-dose lithium supplementation separately alter energy expenditure, body mass, and adipose metabolism for the promotion of thermogenesis

**DOI:** 10.1016/j.isci.2024.109468

**Published:** 2024-03-11

**Authors:** M.S. Finch, G.L. Gardner, J.L. Braun, M.S. Geromella, J. Murphy, K. Colonna, R. Dhaliwal, A. Retta, A. Mohammad, J.A. Stuart, P.J. LeBlanc, V.A. Fajardo, B.D. Roy, R.E.K. MacPherson

**Affiliations:** 1 Department of Health Sciences, Brock University, St. Catharines, ON, Canada; 2 Department of Biological Sciences, Brock University, St. Catharines, ON, Canada; 3 Department of Kinesiology, Brock University, St. Catharines, ON, Canada

**Keywords:** Obesity medicine, Physiology, Cell biology

## Abstract

Nutraceutical approaches to promote adipose tissue thermogenesis may help to prevent obesity onset. Creatine is a critical regulator of adipose metabolic function and low-dose lithium supplementation has been shown to promote adipose thermogenesis. In the present study, we sought to directly compare the two supplements for their effects on adipose metabolism and thermogenesis. We show that both supplements increase daily energy expenditure (EE) and reduce body mass in male Sprague-Dawley rats. Lithium increased brown adipose tissue (BAT) mitochondrial and lipolytic proteins that are associated with thermogenesis, while creatine increased BAT UCP1 and mitochondrial respiration. The BAT thermogenic findings were not observed in females. White adipose tissue and skeletal muscle markers of thermogenesis were unaltered with the supplements. Together, the data show that low-dose lithium and creatine have diverging effects on markers of BAT thermogenesis and that each increase daily EE and lower body mass in a sex-dependent manner.

## Highlights

• Creatine and lithium increase energy expenditure and lower body mass
• Markers of thermogenesis higher with both supplements in male brown adipose
• No thermogenic effect of the supplements on white adipose or skeletal muscle
• Thermogenic effects of the supplements are sex dependent

## Graphical abstract

**Figure:**
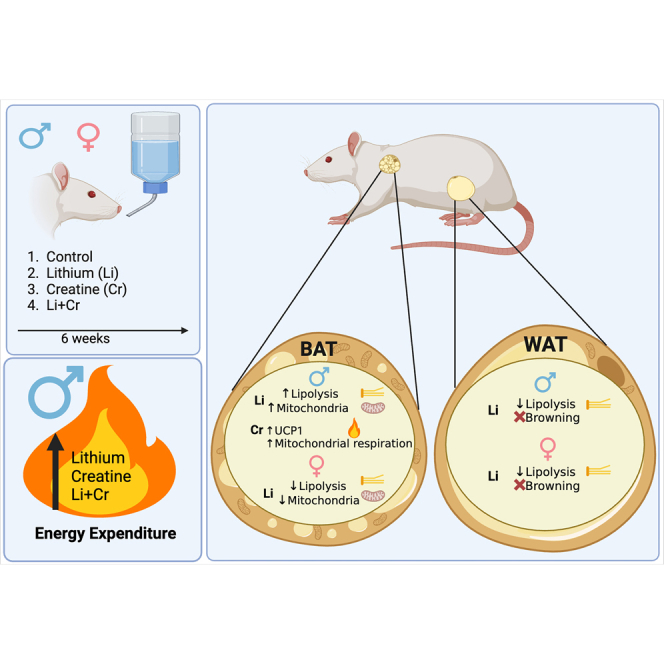

## Introduction

Adipose tissue is classically thought to function as a storehouse for excess energy but has since been recognized for its potential to dissipate energy in the form of heat under mechanisms conserved to combat cold challenges. The process of energy dissipation for heat production in adipose tissue is referred to as nonshivering thermogenesis and in certain circumstances can account for up to 15% of daily energy expenditure in humans.^1^^,^^2^ Promoting the energy expending potential of nonshivering thermogenesis at rest is therefore a valuable target for obesity therapeutics and prophylactics as obesity rates are projected to rise considerably in the coming years.^3^

Lithium supplementation in low doses, resulting in serum concentrations far below the typical therapeutic window for manic disorders (0.5–1.2 mM), has been shown to improve the functional or characteristic markers of many metabolic diseases.^4^^,^^5^ Recently, Geromella et al. demonstrated that low dose lithium supplementation (10 mg/kg/day, resulting in a serum Li concentration of only 0.02 mM^6^) increased daily energy expenditure and promoted a beiging phenotype in inguinal white adipose (iWAT)^7^ in male mice. Lithium has been shown to potently inhibit GSK3^8^ and it is thought that the thermogenesis-inducing properties of lithium are mediated through its inhibition of GSK3. Previous work has uncovered that selective GSK3β inhibition promotes the expression of thermogenesis-associated genes in brown adipocytes *in vitro.*^9^ Furthermore, it is known that GSK3 activity within adipose tissue is 2-fold higher in diet-induced obese mice^10^ and its inhibition can reduce pro-inflammatory M1 macrophages^11^ and improve insulin sensitivity^12^ and signaling.^13^ The previous evidence adds support to the hypothesis that low-dose lithium supplementation can induce positive metabolic outcomes and promote adipose thermogenesis, potentially through its inhibition of GSK3.

Separately, creatine is emerging as a critical regulator of adipose metabolism and thermogenesis. Extensive research has highlighted the crucial roles of endogenous creatine synthesis, the creatine transporter, and the recently discovered futile creatine cycle (FCC) to adipose metabolism and thermogenesis.^14^^,^^15^^,^^16^^,^^17^^,^^18^ However, FCC has only been shown to be activated with adrenergic stimuli which questions its clinical translatability as potent adrenergic stimuli poses many risks for people with obesity.^19^ To our knowledge, it is unknown if creatine supplementation alone can increase markers of adipose thermogenesis at rest. A recent paper has reported that *Ucp1* gene expression was increased with creatine-supplementation in the brown adipose tissue (BAT) of high fat fed (HFD)-fed mice.^20^ In humans, creatine supplementation was unable to increase BAT activity via positron emission tomography (PET) scan^21^ although the dosing of creatine spanned only seven days. Twenty-eight days of creatine supplementation was found to increase resting metabolic rate in humans^22^ but it was not studied if BAT was a contributor to the increase in basal metabolism. Separately, a retrospective study was able to correlate creatinine clearance with BAT activity via PET scan,^23^ which also correlates creatine abundance with thermogenic capacity. Animal studies provide the best evidence for the necessity of available creatine to adipose thermogenic function. Animals treated with a creatine analog that impairs creatine metabolism were found to have less UCP1 in their BAT depot.^24^ In addition, mice that lack the ability to endogenously synthesize creatine within their adipose depots have a reduced whole-body VO_2_ that is recovered by creatine supplementation.^15^ Lastly, work from our lab has observed that creatine supplementation results in higher levels of key mitochondrial markers in adipose across sexes and depots.^25^ The aforementioned supports the hypothesis that creatine availability is a regulator of adipose metabolism and has the potential to influence thermogenic capacity.

The promotion of thermogenesis at rest may be a vital tool to combatting the development of obesity at the population level. The previous evidence suggests that supplemental lithium and creatine could be an effective method of promoting thermogenesis without the risk that comes with administering potent adrenergic stimuli. The aim of the present study is to investigate if lithium and creatine, supplemented separately or concurrently, can increase whole-body energy expenditure (EE) and adipose tissue markers of metabolism and thermogenesis. Importantly, the study also seeks to determine if the proposed thermogenic effects of lithium and creatine supplementation translate to females as all the previously cited evidence in support of lithium and creatine supplementation for promoting adipose thermogenesis was conducted solely in male models. This research will provide evidence for the utility of minimally invasive supplements, when utilized in a chronic manner, to effect energy expenditure and thermogenic markers in adipose tissue. It will also provide sex and depot specific comparisons to determine, which are most affected by the supplementation.

## Results

### Lithium and creatine supplementation resulted in higher energy expenditure and lower body mass in males but not females

**Figure 1:**
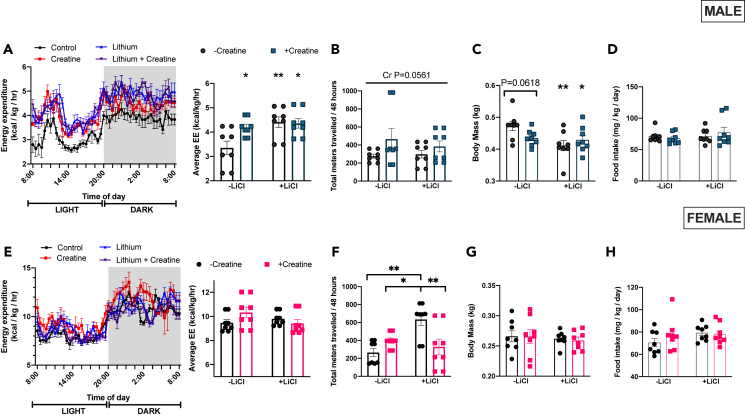
Whole-body parameters (A) Male energy expenditure recorded every 30 min over a 24-h period (left figure) and then averaged over 24-h (right figure) (B) Male total meters traveled in the metabolic caging system as recorded by X, Y, Z beam breaks. (C) Final male body mass in kg. (D) Male food intake was recorded three times weekly and then averaged over the 6-week supplementary period. (E–H) the same measures as A-D but for females. Data were analyzed by two-way ANOVA and are presented as mean ± SEM, ∗ denotes significantly different from control p < 0.05, ∗∗p < 0.01, ∗∗∗p < 0.001, ∗∗∗∗p < 0.0001, p value written above a straight horizontal line is denoting a main effect of its associated treatment.

In males, EE was higher than control in all treatment groups (Figure 1A). The higher EE with Li cannot be attributed to differences in locomotion as total meters traveled within the metabolic caging unit over 48 h were not altered with supplementation (Figure 1B). Similarly, Cr did not increase meters traveled within the caging unit but was approaching significance (p = 0.0561; Figure 1B).

Final body mass was lower than control in Li and Li+Cr and approached significance in Cr (p = 0.0618; Figure 1C). Reductions in body mass are not attributable to reduced food intake as there were no observed differences between groups (Figure 1D).

In females, there was no effect of treatment on EE (Figure 1E). Despite a similar EE, Li animals were found to travel more within their cages compared to all other groups (Figure 1F). There were no effects of treatment on body mass (Figure 1G) or average daily food intake in the females (Figure 1H).

### Creatine increases UCP1, and mitochondrial respiration in male BAT and lithium increase BAT mitochondrial and lipolytic proteins in males but not females

**Figure 2:**
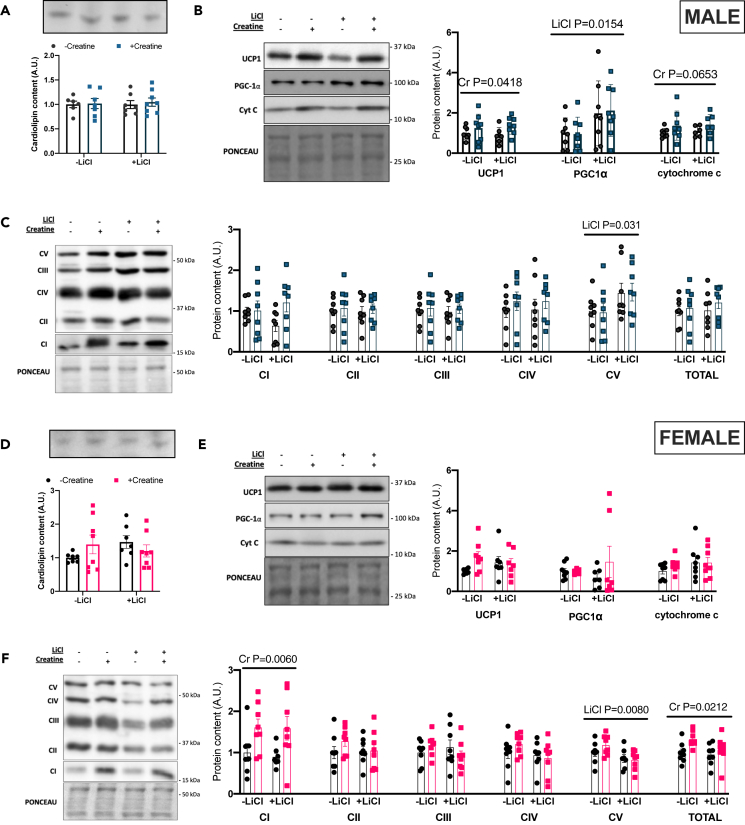
BAT mitochondrial and thermogenic proteins (A) cardiolipin concentration as measured by HPTLC in males. (B) Western blotting quantification for UCP1, PGC1α, and cytochrome *c* in males. (C) Western blotting quantification for OXPHOS antibody cocktail in males. (D) cardiolipin concentration as measured by HPTLC in females. (E) Western blotting quantification for UCP1, PGC1α, and cytochrome c in females. (F) Western blotting quantification for OXPHOS antibody cocktail in females. Data were analyzed by two-way ANOVA and are presented as mean ± SEM, ∗ denotes significantly different from control p < 0.05, ∗∗p < 0.01, ∗∗∗p < 0.001, ∗∗∗∗p < 0.0001, p value written above a straight horizontal line is denoting a main effect of its associated treatment.

To determine if the changes in EE and body mass were a result of increased adipose thermogenesis, thermogenic, mitochondrial, and lipolytic proteins were investigated in BAT. Mitochondrial density, measured as cardiolipin concentration,^26^ was not different between experimental groups in either males (Figure 2A) or females (Figure 2D). Cr but not Li increased protein content of primary thermogenic markers UCP1, and cytochrome *c* which approached significance (p = 0.0653) in males (Figure 2B) with no effect of treatment in females (Figure 2E). Li but not Cr increased PGC1α protein content, a marker of mitochondrial biogenesis, in males but not females (Figures 2B and 2E). Markers of electron transport chain proteins were differentially influenced by the treatments as Complex V was increased in males (Figure 2C) and decreased in females (Figure 2F) with Li treatment. Cr increased Complex I and total protein content of the five complexes of the electron transport chain in females (Figure 2F) but not males (Figure 2C).

**Figure 3:**
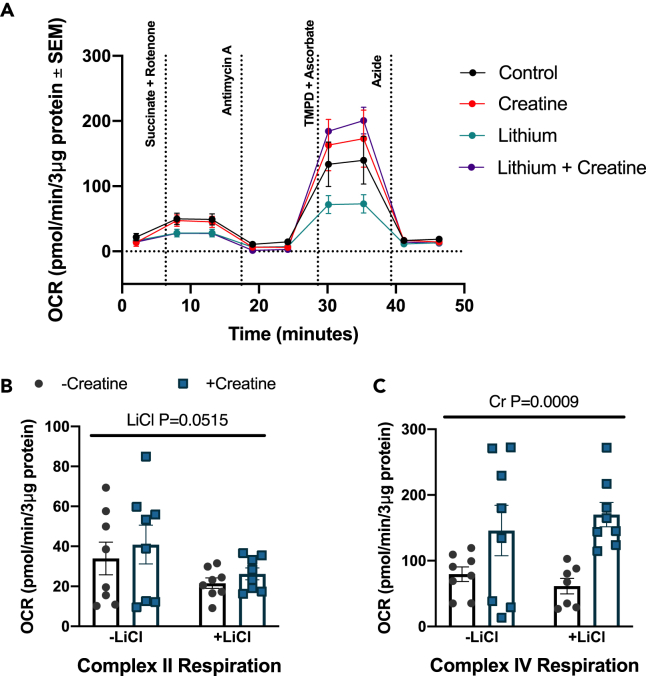
BAT mitochondrial respiration in males (A) Oxygen consumption rate (OCR) over time (B) Complex II respiration (C) Complex IV respiration. p value written above a straight horizontal line is denoting a main effect of its associated treatment. Data are presented as mean ± SEM.

To further examine the potential mechanisms mediating the higher EE in male rats, BAT mitochondrial respiration was examined. No changes were observed in Complex II respiration between treatments (Figures 3A and 3B), Complex IV respiration was higher in Cr- and Li+Cr-treated male BAT (Figures 3A and 3C). Li treatment alone did not affect Complex IV respiration.

**Figure 4:**
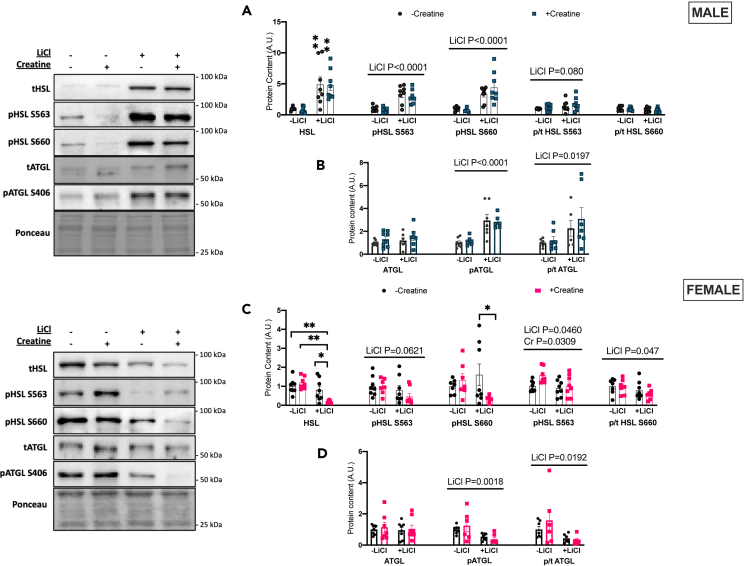
BAT lipolytic proteins (A) Total and phosphorylated HSL (Ser563, Ser660) western blot quantifications for males as well as the ratio of phosphorylated/total protein content for the two above phosphorylation sites (B) Total and phosphorylated ATGL (Ser406) western blot quantifications for males as well as the phosphorylated/total ratio. (C) Total and phosphorylated HSL (Ser563, Ser660) western blot quantifications for females as well as the ratio of phosphorylated/total protein content for the two above phosphorylation sites. (D) Total and phosphorylated ATGL (Ser406) western blot quantifications for females as well as the phosphorylated/total ratio. Data were analyzed by two-way ANOVA and are presented as mean ± SEM, ∗ denotes significantly different from control p < 0.05, ∗∗p < 0.01, ∗∗∗p < 0.001, ∗∗∗∗p < 0.0001, p value written above a straight horizontal line is denoting a main effect of its associated treatment.

Li but not Cr increased lipolytic markers including total hormone sensitive lipase (HSL), phospho-S563 HSL, phospho-S660 HSL (Figure 4A), as well as phospho-S406 ATGL in males (Figure 4B). The ratio of phosphorylated-to-total HSL S660 approached significance (p = 0.080) and the ratio of phosphorylated-to-total ATGL was higher with lithium (Figures 4A and 4B). Conversely, in females, Li and Li+Cr had lower total HSL and phospho-S660 HSL protein whereas Li alone lowered phospho-S563 HSL and phospho-S406 ATGL (Figures 4C and 4D). Li-treated groups also had lower phosphorylated-to-total HSL S563 and S660 ratios as well as the phosphorylated-to-total ATGL ratio in the BAT of females.

### Lithium and creatine supplementation alters WAT adipocyte area

**Figure 5:**
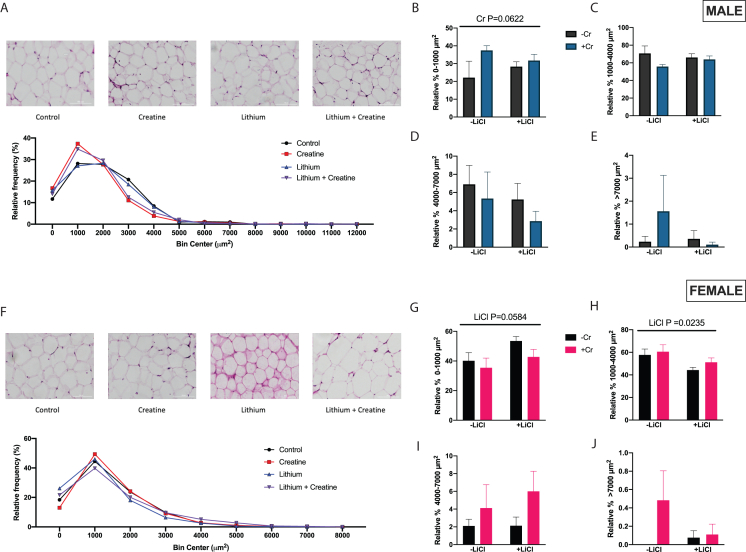
WAT histological characterization (A) H&E-stained adipocytes were quantified for adipocyte area and then put into a histogram for males. (B–E) Adipocytes were grouped into four separate categories: 0–1000 μm^2^, 1100–4000 μm^2^, 4100–7000 μm^2^ and >7000 μm^2^ for males. (F) H&E-stained adipocytes were quantified for adipocyte area and then put into a histogram for females. (G–J) Adipocytes were grouped into four separate categories: 0–1000 μm^2^, 1100–4000 μm^2^, 4100–7000 μm^2^ and >7000 μm^2^ for females. Data were analyzed by two-way ANOVA and are presented as mean ± SEM, ∗ denotes significantly different from control p < 0.05, ∗∗p < 0.01, ∗∗∗p < 0.001, ∗∗∗∗p < 0.0001, p value written above a straight horizontal line is denoting a main effect of its associated treatment. The scale bar on the representative histological images denotes 100μm.

To determine if the treatments shifted WAT morphology toward a beiging phenotype and if this may also contribute to the increased energy expenditure, iWAT was analyzed by H&E stain to assess the presence of multilocular adipocytes and the adipocyte area. There were no visible browning/beiging in either WAT depot between the sexes (Figures 5A and 5F). Despite the lack of overt browning, we decided to examine adipocyte area. In male WAT there was an approaching significant relative frequency shift toward more smaller adipocyte areas in the Cr group (p = 0.0622; Figure 5B). In females, Lithium resulted in less adipocytes in the 1000–4000 μm^2^ range and more adipocytes in the 0–1000 μm^2^ range although the latter only approached significance (p = 0.0584; Figures 5G and 5H).

**Figure 6:**
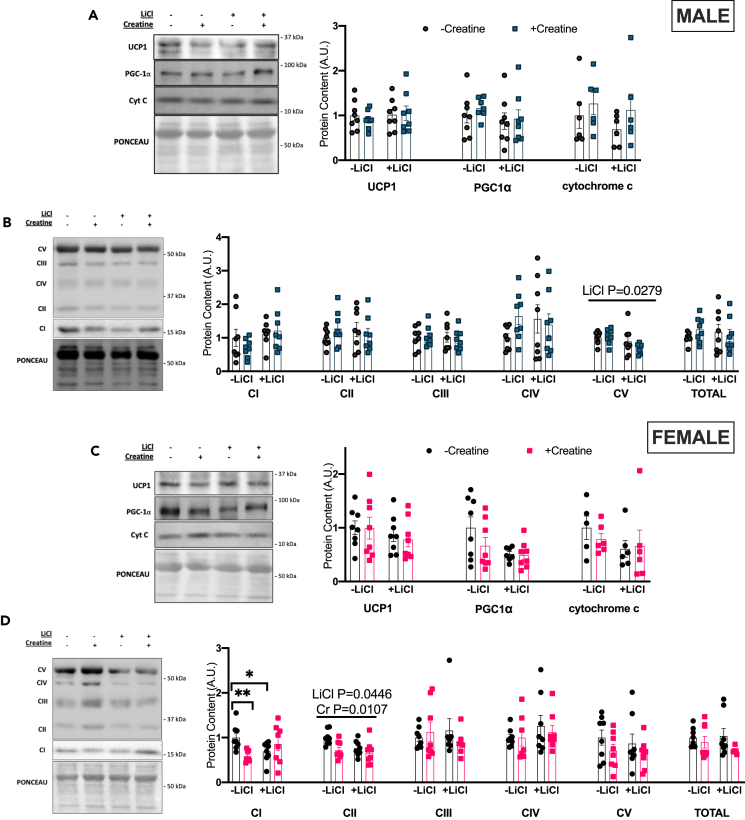
WAT mitochondrial and thermogenic proteins (A) Western blotting quantification for UCP1, PGC1α and cytochrome *c* in males. (B) Western blotting quantification for OXPHOS antibody cocktail in males (C) western blotting quantification for UCP1, PGC1α cytochrome *c* in females (D) western blotting quantification for OXPHOS antibody cocktail in females. Data were analyzed by two-way ANOVA and are presented as mean ± SEM, ∗ denotes significantly different from control p < 0.05, ∗∗p < 0.01, ∗∗∗p < 0.001, ∗∗∗∗p < 0.0001, p value written above a straight horizontal line is denoting a main effect of its associated treatment.

To further examine the effects of the supplements on adipose thermogenesis, markers of thermogenesis were examined in WAT. Both Li and Cr had no effect on UCP1, PGC1α or cytochrome *c* protein content in the WAT of either sex (Figures 6A and 6C). At the level of the electron transport chain, Li decreased protein content of Complex V in males (Figure 6B) whereas Li and Cr independently decreased Complex I and II in females (Figure 6D).

### WAT lipolytic markers are lower with lithium-supplementation in both sexes

**Figure 7:**
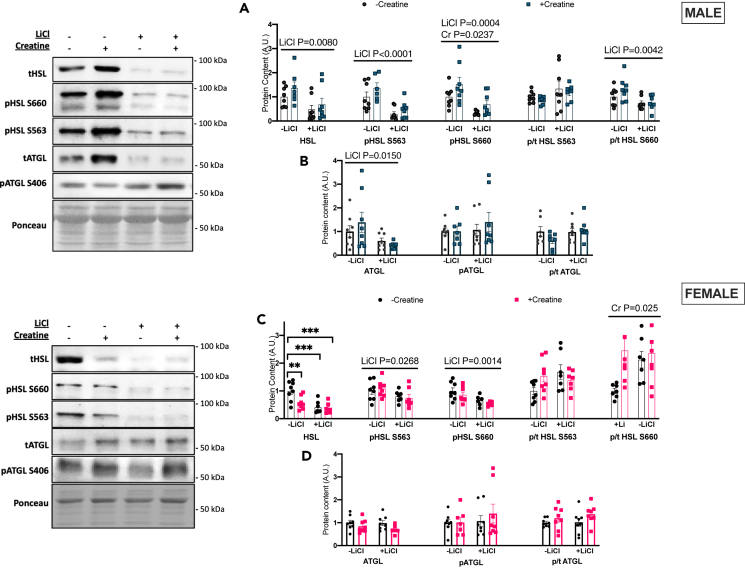
BAT lipolytic proteins (A) Total and phosphorylated HSL (Ser563, Ser660) western blot quantifications for males as well as the ratio of phosphorylated/total protein content for the two above phosphorylation sites. (B) Total and phosphorylated ATGL (Ser406) western blot quantifications for males as well as the phosphorylated/total ratio. (C) Total and phosphorylated HSL (Ser563, Ser660) western blot quantifications for females as well as the ratio of phosphorylated/total protein content for the two above phosphorylation sites. (D) Total and phosphorylated ATGL (Ser406) western blot quantifications for females as well as the phosphorylated/total ratio. Data were analyzed by two-way ANOVA and are presented as mean ± SEM, ∗ denotes significantly different from control p < 0.05, ∗∗p < 0.01, ∗∗∗p < 0.001, ∗∗∗∗p < 0.0001, p value written above a straight horizontal line is denoting a main effect of its associated treatment.

Finally, Li decreased lipolytic proteins including total HSL, phospho-S660 HSL, phospho-S563 HSL, and total ATGL in males as well as the phosphorylated-to-total HSL S660 ratio (Figures 7A and 7B). The lithium effects on lipolytic markers continued to phospho-S563 HSL and phospho-S660 HSL in females being lower with lithium (Figure 7C) with no effect of the treatments on total or phosphorylated ATGL protein content (Figure 7D). In contrast, creatine-treated groups were found to have higher phospho-S660 HSL in males and higher phosphorylated-to-total HSL S660 ratio (Figures 7A and 7C).

### Lithium increased GSK3β Ser9 inhibitory phosphorylation in BAT in males but not females with major sex differences in BAT GSK3 content

**Figure 8:**
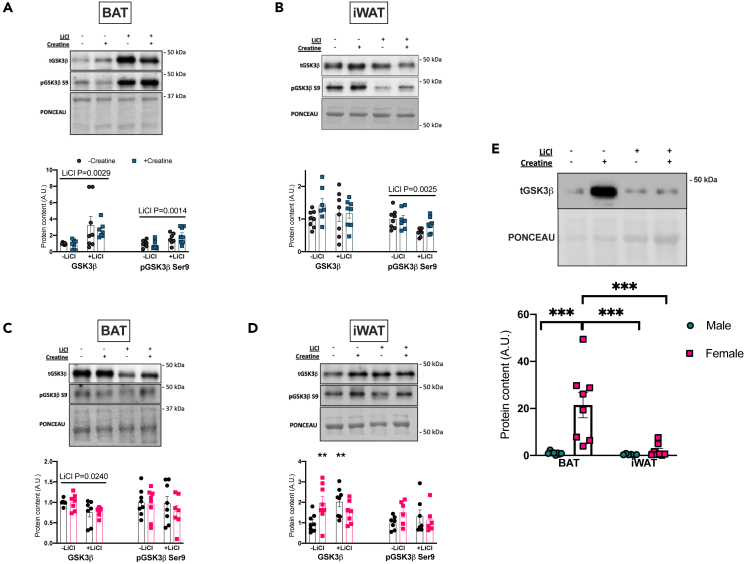
GSK3β content and inhibitory phosphorylation in BAT and iWAT (A) Total and Ser9 phosphorylated GSK3β western blot quantification in male BAT. (B) Total and Ser9 phosphorylated GSK3β western blot quantification in male WAT. (C) Total and Ser9 phosphorylated GSK3β western blot quantification in female BAT. (D) Total and Ser9 phosphorylated GSK3β western blot quantification in female iWAT. (E) Total GSK3β protein content relative to total protein content equalized across male and female brown and white adipose tissue to compare relative protein expressions across tissues. Data were analyzed by two-way ANOVA and are presented as mean ± SEM, ∗ denotes significantly different from control p < 0.05, ∗∗p < 0.01, ∗∗∗p < 0.001, ∗∗∗∗p < 0.0001, p value written above a straight horizontal line is denoting a main effect of its associated treatment.

Li increased total and phospho-S9 GSK3β in BAT of males (Figure 8A) whereas total GSK3β in females was lower with Li and had no effect on GSK3β S9 phosphorylation (Figure 8C). In iWAT, Li decreased phospho-Ser9 GSK3β in males (Figure 8B). In contrast, Li and Cr increased total GSK3β with no change in phosphorylated GSK3β in females (Figure 8D). Lastly, it was found that total GSK3 protein content was ∼20-fold higher in female BAT compared to males (Figure 8E) and may help to explain the sexually dimorphic effects of lithium on markers of BAT metabolism.

## Discussion

### Alterations to brown adipose metabolism may underlie the sex specific whole-body effects of the supplements

It is important to study the chronic administration of nutritional supplements for their utility in promoting thermogenesis as a prophylactic measure to combat the rising rates of obesity in the developed world.^27^ This study sought to compare the adipose depot effects of two nutritional supplements, creatine and lithium, which we have previously shown to exert effects on adipose thermogenesis. We demonstrate that lithium and creatine supplementation similarly increase EE and lower body weight in male rats consistent with what would be seen with increased nonshivering thermogenesis. We then identified alterations in thermogenic, mitochondrial, and lipolytic markers in BAT with minimal effects on skeletal muscle and WAT markers of thermogenesis. This would suggest that both creatine and lithium exert their thermogenic effects mostly via alterations to BAT but diverge in the markers altered by each individual supplement.

Creatine was found to increase Complex IV respiration and UCP1 and cytochrome *c* protein content in male BAT. In concordance with this finding, a recent study has shown an increased *Ucp1* gene expression with creatine supplementation in rodents.^20^ Furthermore, it was found in a model of chronic creatine depletion that UCP1 protein expression is reduced following beta-guanidinopropionic acid treatment, an inhibitor of cellular creatine uptake.^24^ Our Complex IV respiration and cytochrome *c* protein expression findings, taken together with UCP1 expression, are suggestive of thermogenic mitochondrial uncoupling in male BAT following creatine treatment. Increased electron transport chain activity likely results from UCP1 uncoupling, with futile respiratory activity contributing to increased energy expenditure observed in male mice.

Lithium increased markers of lipolysis in the BAT of males, along with PGC1α and Complex V protein expression. It is the GSK3 inhibition by lithium that we believe to be a key to the effects of lithium on BAT. It is known that inhibitory phosphorylation of GSK3 can upregulate mitochondrial function.^28^ PGC1α was found to be higher with lithium supplementation and could be secondary to lithium-mediated GSK3-inibition.^29^^,^^30^ There were, however, no changes in cardiolipin content suggesting the higher PGC1α content did not induce mitochondrial biogenesis in this model. It is known that GSK3 regulates mitochondrial functions that go beyond biogenesis and could still be mediated by PGC1α.^28^ It is also possible that the higher PGC1α that was observed with lithium supplementation could be a result of the higher lipolytic markers in the depot as the activation of lipolysis results in increased activity of transcription factors involved in thermogenic remodeling, including PGC1α.^31^ Total and S563 and S660 phosphorylated HSL and phosphorylated ATGL were higher in lithium supplemented groups, which was also true of the ratios of phosphorylated-to-total protein content for both proteins in male BAT. The opposite was observed in female BAT and aligns well with the data suggesting that GSK3 is more inhibited at its Ser9 site in male BAT but not female BAT.

Together, the data suggest that creatine and lithium supplementation both exert effects on BAT thermogenic markers, however, the markers that are altered diverge between the supplements. Creatine was found to increase more typical UCP1-dependent nonshivering thermogenesis at the BAT, whereas lithium increased markers of lipolysis which could suggest a GSK3-lipolysis induced mechanism of thermogenesis that is not present in creatine-treated animals. As there were no synergistic effects of the supplements, it is likely that these separate mechanisms of thermogenesis cannot be concurrently activated. It has been shown previously that UCP1-dependent thermogenesis will be active at the expense of UCP1-independent mechanisms^14^ but more research into this topic field is needed before such a hypothesis can be applied to the present work. It will be important in the future to determine dose-response curves for the supplements to determine optimal supplementation paradigms based on circulating concentrations and are a limitation of the present study.

### Creatine and lithium exert sex-specific effects on markers of thermogenesis

Our lab has previously shown that a similar creatine supplementation protocol increases creatine content in the BAT of males with no difference in females.^25^ This difference in males and females could underlie the sex-specificity of the present creatine-induced UCP1 and energy expenditure findings. A sex-dimorphism in the effects of creatine supplementation have been repeatedly observed and requires further examination.^32^^,^^33^ One possible underlying mechanism behind the sex difference in creatine effect is that females have 10% greater resting intramuscular creatine stores.^34^ Since brown adipocytes and myocytes are of the same developmental origin, it is possible that female BAT has greater resting creatine stores and therefore supplementation would have a lesser effect on intracellular supply but requires further examination and the utilization of estrogen-depleted animal models to determine the estrogen-dependence of this observation.

An important difference that may underlie the sex specificity of the lithium results is the GSK3β response to supplementation. In male BAT, lithium supplementation resulted in higher GSK3β phosphorylation at its Ser9 inhibitory site, as initially hypothesized, and this effect is absent in females. The cause of the sex-specific effect of lithium supplementation is unknown but one possibility is that the females have higher GSK3β protein content and since the kinase is constitutively active, it may be harder for a low dose of lithium to have a strong inhibitory effect on GSK3β.

### WAT and skeletal muscle do not take on thermogenic characteristics with the supplements

WAT can express functionally thermogenic proteins that are upregulated in a process known as browning/beiging and contribute to daily EE.^35^ We therefore examined the influence of lithium and creatine on WAT morphology and thermogenic protein expression. There were minimal effects of both treatments on WAT adipocyte area across sexes including no observed multilocularity and therefore no overt adipocyte browning/beiging effect. There were minor changes observed in ETC proteins and were independent of any changes in the primary thermogenic proteins UCP1 and PGC1α. Lipolytic markers were also lower in WAT of lithium treated groups in both sexes. Collectively, these results suggest that the treatments are specific to BAT for their promotion of thermogenic markers. And contrasts with the study by Geromella et al.^36^ which observed a consistent increase in mitochondrial proteins, including UCP1, and multilocular phenotype in WAT with low-dose lithium supplementation. The difference in results is likely due to model differences as the present study was done in rats compared to a mouse model used in the previous study. The two studies are similar, however, in that it is the depot in which GSK3β is more inhibited by lithium that will result in changes to thermogenesis associated proteins.

Skeletal muscle is also a contributor to nonshivering thermogenesis and has the potential to increase daily energy expenditure,^37^^,^^38^ therefore we also included examination of skeletal muscle nonshivering thermogenesis. Skeletal muscle nonshivering thermogenesis is thought to function primarily through the inefficient transport of calcium at the sarco(endo)plasmic reticulum Ca^2+^ ATPase (SERCA).^39^^,^^40^ Under optimal conditions SERCA transports two calcium ions into the sarcoplasmic reticulum per ATP molecule hydrolyzed,^41^ however, this coupling ratio is reduced in the presence of SERCA uncouplers sarcolipin (SLN) and neuronatin (NNAT).^42^^,^^43^^,^^44^ Low-dose lithium supplementation has been shown previously to increase SERCA uncoupling in C2C12 myoblasts and in the soleus of chow and HFD-fed mice. Similar uncoupling and upregulation of SERCA uncouplers was observed in muscle-specific GSK3 knockdown mice.^36^ In the present study we examined SERCA coupling along with the protein expression of known uncouplers and contributors to SERCA-dependent thermogenesis including GSK3, RYR, SLN, and NNAT. None of the previous markers of muscle nonshivering thermogenesis were altered with lithium supplementation (Figure S1) and may be dissimilar to previous results due to the utilization of a different rodent model and dosing than was used previously. The changes in energy expenditure with the treatments is therefore likely coming from the adipose tissue.

Increasing thermogenic activity at rest with nutritional supplements might be a particularly useful prophylactic tool to mitigate the likelihood of obesity development as present prophylactic obesity tools have proven either dangerous or ineffective. The results of the present study highlight the potential utility of low-dose lithium and creatine supplementation to impact EE, body mass, and markers of adipose thermogenesis, however, this was primarily in the BAT depot and there was no synergy detected between the two supplements, highlighting distinct and separate modes of action. Future work could test the effects of increasing the dose of lithium especially given that our study is limited in that we could not measure serum Li levels. Furthermore, as the thermogenic effects of these two supplements were only found in male rats, it would be of great importance to determine why the results varied between male and female rats as it will pave a path toward making these supplements effective for thermogenesis in both sexes. In this respect, focusing on the effects of sex hormones and their possible interplay with these supplements would likely shed valuable insight.

### Limitations of the study

The present study was limited in that the serum concentrations of lithium and creatine were not recorded. However, it has been previously shown that the same creatine supplementation protocol used in the present study results in increased adipose tissue creatine content suggesting that a suitable creatine supplementation protocol was selected.^25^ Similarly, the serum concentration of lithium in the present study is unknown but is expected to be ∼0.1 mM based on previous literature, which is far below typical therapeutic values of ∼1.2 mM^45^ and is similar to studies that show thermogenic potential for lithium.^36^ Furthermore it is unknown if the sex-specificity of the findings are estrogen-dependent or are resultant of another mechanism. It is of critical importance to determine how to overcome sex-dependent mechanistic differences to promote optimal supplementation protocols for both sexes. Lastly, the supplements were provided *ad libitum* to the animals in their water supply and therefore the amounts of the supplements consumed between animals is variable. Natural variability in dosing can result in the inability to capture minute effects in outcome variables, however, many robust changes were observed despite the variability in dosing, which can be interpreted as strong effects of the supplements that persist despite the variability in dosing.

## STAR★Methods

### Key resources table

**Table:** 

REAGENT or RESOURCE	SOURCE	IDENTIFIER
**Antibodies**		

phosphorylated HSL Ser563	Cell Signaling	Cat#4139; RRID: AB_2135495
HSL (Ser660 phosphorylated)	Cell Signalling	Cat# 4126; RRID: AB_490997
HSL	Cell Signalling	Cat# 4107; RRID: AB_2296900
ATGL (Ser406 phosphorylated)	Abcam	Cat# ab135093; RRID: AB_2888660
ATGL	Cell Signaling	Cat# 2439; RRID: AB_2167953
UCP1	Abcam	Cat# ab10983; RRID: AB_2241462
PGC1α	Milipore	Cat# AB3242; RRID: AB_2268462
Cytochrome c	Abcam	Cat# ab76237; RRID: AB_1523454
OXPHOS antibody cocktail	Abcam	Cat# ab110413; RRID: AB_2629281
GSK3β (Ser9 phosphorylated)	Cell Signaling	Cat# 5558; RRID: AB_10013750
GSK3β	Cell Signaling	Cat# 9315; RRID: AB_490890
Donkey Anti-Rabbit	Jackson Immunoresearch Laboratories	Cat# 711-035-152; RRID: AB_10015282
Goat Anti-Mouse	Jackson Immunoresearch Laboratories	Cat# 115-035-003; RRID: AB_10015289
phosphorylated HSL Ser660	Cell Signaling	Cat#4126; RRID: AB_490997
HSL	Cell Signaling	Cat#4107; RRID: AB_2296900
phosphorylated ATGL Ser406	Abcam	Cat#ab135093; RRID: AB_2888660
ATGL	Cell Signaling	Cat#2439; RRID: AB_2167953
UCP1	Abcam	Cat#ab10983; RRID: AB_2241462
PGC1α	Milipore	Cat#AB3242; RRID: AB_2268462
Cytochrome c	Abcam	Cat#ab76237; RRID: AB_1523454
OXPHOS antibody cocktail CAT# ab110413; RRID: AB_2629281	Abcam	Cat#ab110413; RRID: AB_2629281
phosphorylated GSK3β Ser9	Cell Signaling	Cat#5558; RRID:AB_10013750
GSK3β	Cell Signaling	Cat#9315; RRID: AB_490890

**Experimental models: Organisms/strains**		

Rat : Sprague Dawley : wild type	Charles River	N/A

**Software and algorithms**		

Graphpad Prism 8	Graphpad Prism	https://www.graphpad.com/scientific-software/prism/
ImageJ	NIH	https://imagej.nih.gov/ij/download.html
AlphaView	ProteinSimple	https://alphaview-sa1.software.informer.com/
Prometion 30-minute Rat Macro	Sable Systems	https://www.sablesys.com/products/promethion-high-definition-room-calorimetry-system/promethion-software/

### Resource availability

#### Lead contact

Further information and requests for resources and reagents should be directed to and will be fulfilled by the lead contact, Rebecca E.K. MacPherson (rmacpherson@brocku.ca).

#### Materials availability

This study did not henerate new unique reagents.

#### Data and code availability

• All data reported in this paper will be shared by the lead contact upon request.
• This paper does not report original code.
• Any additional information required to reanalyze the data reported in this paper is available from the lead contact upon request.

### Experimental model and study participant details

#### Animals

Sixty-four 6-week-old Sprague-Dawley Rats (32 males and 32 females) were randomly assigned into 4 groups: control (Con), lithium (Li), creatine (Cr), and combination of lithium and creatine supplementation (Li+Cr). The treatment groups received either 5 g/L of creatine monohydrate (SIGMA cat #84147000), 200 mg/L lithium chloride (LiCl, SIGMA cat#L9650), or concurrent treatment of LiCl and Cr in their drinking water for 6 weeks. The lithium dosing was formulated to acheive an expected serum lithium concentration of ∼0.1 mM, based on previous literature in which a 600 mg/L dosing via the drinking water resulted in serum lithium concentration of 0.260 mM,^45^ and is similar to the dosage in which we previously found increased markers of adipose thermogenesis with lithium treatment.^36^ The dosing of creatine was chosen from our previous work that showed that the same creatine dose leads to higher adipose creatine content.^25^ All treatment groups received 1% sucrose in their water supplies for palatability. Water solutions were refreshed every two days. *Ad libitum* access to chow pellets (AIN-93G, Envigo) was available to the animals and food and water intakes along with body mass were recorded 3 times per week until endpoint (6 weeks after treatment began).

#### Ethics statement

The animals were kept in a 12:12 light-dark cycle. Experimental procedures were approved by the Brock University Animal Care Committee and were in compliance with the Canadian Council on Animal Care.

### Method details

#### Metabolic caging

On the final week of treatment intervention, the animals were placed in Sable Systems Promethion metabolic cages (Sable Systems International, Las Vegas, NV) on a 12-hour light/dark cycle for 48 hours. Oxygen consumption, EE in kcal/hr, and total meters travelled were continuously recorded and averaged into 30-minute intervals using Sable Systems data acquisition software. Data were analyzed using Sable Systems International Macro Interpreter software (v.2.48) using One-Click Macro.^36^

#### Tissue collection

The animals underwent non-survival surgeries using isoflurane gas anesthesia. WAT samples were collected from the inguinal subcutaneous depot and BAT samples were collected from the interscapular fat pads. Soleus muscle was also collected at this time. Adipose samples were divided and placed in either formalin for histological analysis or snap frozen in liquid nitrogen and stored at −80°C for analysis via Western blotting.

#### Western blotting

Adipose tissue samples were homogenized using a FastPrep-FP120 Tissue Homogenizer (Savant) in a 1:3 ratio of sample in mg to μL of lysis buffer (10 ml NP40 lysis buffer, 34μL phenylmethane sulfonyl fluoride and 50μL protease inhibitor) for white adipose depots and a 1:5 ratio for BAT. The homogenized sample was centrifuged at 4°C for 5 minutes at 1500 x g and the middle aqueous layer was collected, pellet and upper lipid layer were discarded. Bicinchoninic acid assay (BCA) was utilized to determine protein concentration. The samples were prepared to contain equal concentrations of protein in 2x Laemmli buffer and were linearized at 100°C for 5 minutes. All BAT samples were loaded at 5 μg of protein, 10-20 μg of protein were loaded for iWAT, except for UCP1 in which 30 μg was loaded onto 10% SDS-PAGE gels and ran for 90 minutes at 120V and subsequently wet transferred onto 0.45 μm nitrocellulose membranes. Membranes were stained with Ponceau S Solution (Bioshop) to ensure even protein loading and then removed with TBST (tris-buffered saline/0.1% tween 20) washing (3 x 5min). Resulting membranes were blocked in 5% non-fat dry milk-TBST (tris-buffered saline/0.1% tween 20) for 1 hour and incubated overnight at 4°C in primary antibody at 1:1000 concentration. Membranes were rinsed with TBST and incubated in the appropriate secondary antibody (1:5000 concentration for donkey anti-rabbit, 1:10,000 for goat anti-mouse) for 1 hour at room temperature then washed again and imaged. Primary antibodies include: phosphorylated HSL Ser563 (Cell Signaling CAT# 4139), phosphorylated HSL Ser660 (Cell Signaling CAT# 4126), total HSL (Cell Signaling CAT# 4107), phosphorylated ATGL Ser406 (Abcam CAT# ab135093), total ATGL (Cell Signaling CAT# 2439), UCP1 (Abcam CAT# ab10983), PGC1α (Milipore CAT# AB3242), cytochrome c (Abcam CAT# ab76237), OXPHOS antibody cocktail (Abcam CAT#ab110413), phosphorylated GSK3β Ser9 (Cell Signaling CAT# 5558) and total GSK3β (Cell Signaling CAT# 9315). Images were captured on the BioRad ChemiDoc touch imaging system (CAT# 1708370) using Western Lightning ECL western blot substrate (CAT# NEL103E001EA). Analysis of western blot images was performed on AlphaView (ProteinSimple) software. Data was normalized to total protein loading (Ponceau S stain) and made relative to the control group to observe changes in the treatment group as a fold-change compared to control.

#### Histology

Samples were fixed in 10% neutral buffered formalin (Millipore Sigma, CAT#HT501128) for 48 hours and then stored in 70% ethanol. Samples were then dehydrated in ethanol (1 × 90% 30 min, 3 × 100% 40 min) and xylene (Fischer Scientific) (3 × 45 min), embedded in paraffin, 10 μm sections were cut (Rotary Microtome HM 325, Thermo Scientific) and mounted on slides. Slides were stained with Harris hematoxylin and eosin (H&E),^46^ and then imaged using a BioTek Cytation 5 Cell Imaging Multi-Mode Reader (1321000). Three representative images were taken for each sample (∼150 cells/image). The mean cross-sectional area and percent multilocular were analyzed using ImageJ (ImageJ software, National Institute of Mental Health, Bethesda, MD, USA). Adipocyte areas were analyzed via histogram and then the sum and average relative frequencies categorized into four bins: 0-1000 μm^2^, 1100-4000 μm^2^, 4100-7000 μm^2^ and >7000 μm^2^.^47^

#### Mitochondrial respiration in frozen tissue

Methods for measuring mitochondrial respiration in frozen BAT using Seahorse analysis were adapted from previously published methods papers.^48^^,^^49^ For each biological sample, 10mg of brown adipose tissue was thawed on ice and transferred to a pre-chilled small volume glass-to-glass Dounce homogenizer. Samples were homogenized using 500 uL ice-cold 1 x MAS buffer (70 mM sucrose, 220 mM mannitol, 5 mM KH2PO4, 5 mM MgCl2, 1 mM EGTA, 2 mM HEPES pH 7.4) per 10mg sample, and twenty manual strokes of the tissue homogenizer. Homogenates were centrifuged at 2000 x g for 3 minutes at 4°C and the supernatant collected. Protein concentration was determined using a BCA. Using the BCA protein concentrations, homogenates were diluted in MAS buffer to normalize samples to 3 ug sample protein per well in 60uL of sample (0.05ug/uL). Homogenate dilutions were then loaded in triplicate per biological sample to a pre-warmed Seahorse XFe24 microplate and centrifuged at 2,000 x g for 5 min using a plate centrifuge (low break). 390uL of pre-warmed 1 x MAS buffer (pH 7.4) supplemented with 10ug/mL (final concentration) was then gently added to each well. The plate was immediately loaded into a XFe24 Analyzer pre-calibrated with XFe24 cartridge sensors. Substrate injections (final concentrations) for the measurement of respiratory capacity through complex II and IV were as follows: Port A) 5mM succinate and 2uM rotenone, Port B) 4uM Antimycin A, Port C) 0.5mM TMPD and 1mM ascorbic acid, Port D) 50mM sodium azide. Following each injection, oxygen consumption rate (OCR) was measured for 4 minutes two times, with a 30 second mix period allowed before each measurement period. OCR data was exported from the Wave software (Agilent) with no further normalization required due to the normalization of samples to 3ug protein prior to loading. All OCR were thus exported as pmol/min/3ug protein. Complex II- and IV—dependent respiration was calculated by subtracting OCR following complex inhibitor injection from OCR following substrate injection.

#### Cardiolipin concentration analysis with HPTLC

Total lipids were extracted from 50ug of frozen BAT tissue as previously described.^50^ The lipid extract was spotted onto high-performance thin layer chromatography plates (HPTLC; 5633-5, EMD Chemicals, Darmstadt, Germany) and neutral lipids were separated from phospholipids using hexane/diethyl ether/acetic acid (70:30:1, by vol.) as a solvent^51^ after allowing the solvent to run up each plate for 30 min. The phospholipid band was scrapped from the HPTLC plate, lipid extracted again, and spotted onto another HPTLC plate, this time using chloroform:methanol:acetic acid:water (100:75:7:4, by vol.) as a solvent system to separate individual phospholipids,^52^ allowing the solvent to run up each plate for 30 min. The plates were then charred at 180°C after saturation with a 50% aqueous sulfuric acid solution. Cardiolipin was quantified by obtaining densitometric images using the BioRad ChemiDoc touch imaging system (CAT# 1708370) and quantification performed on AlphaView (ProteinSimple) software.

### Quantification and statistical analysis

#### Statistical analysis

Comparisons across treatments were performed via two-way ANOVA presented as mean ± SEM with all measurements being made relative to the control groups. Post-hoc analysis was completed with Fischer’s multiple comparisons test. Statistical significance was assumed at p ≤ 0.05. GraphPad Prism 8 software (GraphPad Software, La Jolla, CA, USA) was used to perform all statistical analyses. ∗ denotes significantly different from control P< 0.05, ∗∗ P<0.01, ∗∗∗ P<0.001, ∗∗∗∗ P<0.0001, P value written above a straight horizontal line is denoting a main effect of its associated treatment.
