# Supplementary material for: Creatine and low-dose lithium supplementation separately alter energy expenditure, body mass, and adipose metabolism for the promotion of thermogenesis
Source: iScience. 2024 Mar 11;27(4):109468. doi: 10.1016/j.isci.2024.109468 (PMC10973579; doi:10.1016/j.isci.2024.109468)
Supplement: Document S1. Figure S1 and Tables S1 and S2 [file mmc1.pdf]

**iScience, Volume *27***

## Supplemental information

# Creatine and low-dose lithium supplementation separately alter energy expenditure, body mass, and adipose metabolism for the promotion of thermogenesis

**M.S. Finch, G.L. Gardner, J.L. Braun, M.S. Geromella, J. Murphy, K. Colonna, R. Dhaliwal, A. Retta, A. Mohammad, J.A. Stuart, P.J. LeBlanc, V.A. Fajardo, B.D. Roy, and R.E.K. MacPherson**

**Supplementary Table 1: Average daily lithium intake (mg / kg / day) averaged over 6-week supplementary period, related to STAR methods: Experimental model and study participant detail.**

| | MALE | FEMALE |
|---|---|---|
| **Li** | 20.27 ± 4.05 | 23.40 ± 3.20 |
| **Li + Cr** | 16.80 ± 1.53 | 16.99 ± 2.22 |

**Supplementary Table 2: Average daily creatine intake (mg / kg / day) averaged over 6-week supplementary period, related to STAR methods: Experimental model and study participant detail. related to STAR methods: Experimental model and study participant detail.**

| | MALE | FEMALE |
|---|---|---|
| **Cr** | 539.94 ± 35.81 | 579.18 ± 87.49 |
| **Li + Cr** | 355.97 ± 70.10 | 424.67 ± 55.56 |

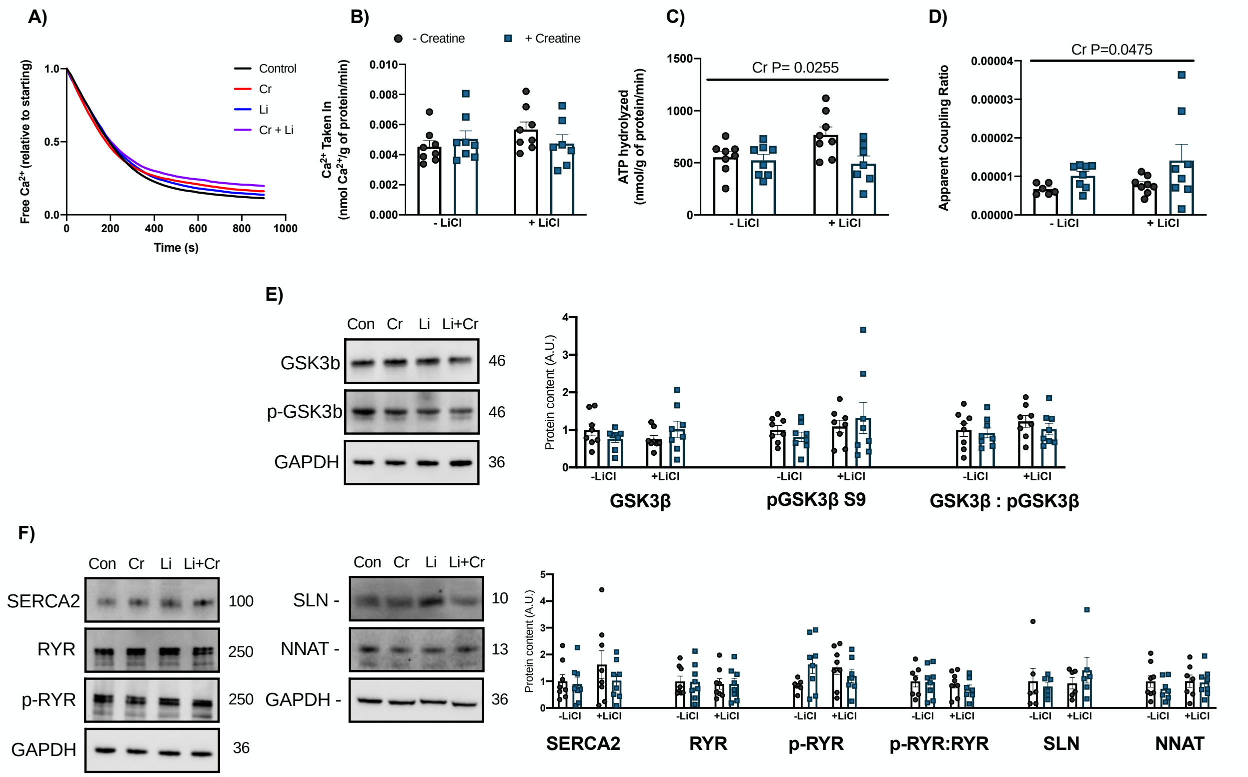


Supplementary Figure 1: Quantification of SERCA function and uncouplers in skeletal muscle, related to STAR methods: Method details. SERCA coupling and protein expression of known uncouplers in the male soleus. A) Free calcium plotted over time B) Calcium per gram of protein per minute C) ATP hydrolyzed per gram of protein per minute D) apparent coupling ratio of ATP hydrolyzed to calcium taken into the sarcoplasmic reticulum E) Western blots of GSK3β and serine 9 phosphorylated GSK3β and the ratio from of total to phosphorylated GSK3β F) Western blots of SERCA, ryanodine receptor, phosphorylated ryanodine receptor Ser 2843, phospho-to-total RYR ratio, sarcolipin and neuronatin.
